# Impact of COVID−19 pandemic on neurodevelopmental outcome in very low birth weight infants: a nationwide cohort study

**DOI:** 10.3389/fped.2024.1368677

**Published:** 2024-09-05

**Authors:** Hyuna Kim, Yong Hun Jang, Joo Young Lee, Gang Yi Lee, Jae Yong Sung, Mi Jung Kim, Bong Gun Lee, Seung Yang, Jinsoo Kim, Kyung Seu Yoon, Ja-Hye Ahn, Hyun Ju Lee

**Affiliations:** ^1^ Department of Translational Medicine, Hanyang University Graduate School of Biomedical Science and Engineering, Seoul, Republic of Korea; ^2^ Department of Medicine, Hanyang University College of Medicine, Seoul, Republic of Korea; ^3^ Department of Rehabilitation Medicine, Hanyang University College of Medicine, Seoul, Republic of Korea; ^4^ Department of Orthopedic Surgery, Hanyang University College of Medicine, Seoul, Republic of Korea; ^5^ Department of Pediatrics, Hanyang University Hospital, Hanyang University College of Medicine, Seoul, Republic of Korea; ^6^ Department of Emergency Medicine, Hanyang University Seoul Hospital, Hanyang University College of Medicine, Seoul, Republic of Korea; ^7^ Department of Psychiatry, Hanyang University Medical Center, Seoul, Republic of Korea; ^8^ Hanyang Institute of Bioscience and Biotechnology, Hanyang University, Seoul, Republic of Korea

**Keywords:** COVID-19, neurodevelopmental outcome, very low birth weight infants, preterm, Korean Neonatal Network

## Abstract

**Introduction:**

Children who have experienced the coronavirus disease 2019 (COVID-19) pandemic are at an increased risk of adverse neurologic developmental outcomes. Limited data exist on the environmental influences of during the COVID-19 pandemic on preterm infant development. This study aimed to investigate whether COVID-19 exposure affects the neurodevelopmental outcomes in preterm children up to 3 years of age.

**Methods:**

This prospective cohort study included all very low birth weight (VLBW) infants from the Korean Neonatal Network who had undergone a neurodevelopmental assessment between January 2015, and May 2022. The neurodevelopmental outcomes along with the scores on the Bayley Scales of Infant and Toddler Development (BSID) and the Korean Developmental Screening Test for Infants and Children of pediatric patients aged 18–24 and 33–39 months who were exposed to COVID-19 were compared with those of VLBW children born and tested before the pandemic.

**Results:**

The cohort included 1,683 VLBW infants. The pandemic group had significantly lower language scores on the BSID-III at ages 18–24 months (*p *= 0.021) and 33–39 months (*p *= 0.023) than the pre-pandemic group after adjusting for gestational age, morbidity, and environmental factors. At 2nd follow-up period, the pandemic group showed significantly lower scores in the cognitive (*p *= 0.026) domains with a mean difference of 7 points and had a significantly higher percentage of ≤−1SD in the gross motor domain (*p *< 0.001) compared with the pre-pandemic group.

**Conclusion:**

Preterm children who experienced the COVID-19 pandemic are at higher risk of abnormal neurodevelopmental outcomes in the first 3 years of life than preterm infants born before the COVID-19 pandemic.

## 1 Introduction

Severe acute respiratory syndrome coronavirus 2, which caused coronavirus disease 2019 (COVID-19), initially emerged in Wuhan, China, in December 2019 and was reported in Korea on January 20, 2020. As the virus primarily spreads through contact with droplets and inhalation of aerosol particles, wearing of face masks and maintaining social distancing should be strictly observed (1). Additionally, the stringent implementation of containment strategies, such as restrictions on the size of gatherings and reductions of outdoor activities, has progressively deteriorated interpersonal relationships (2). The necessity of short- and long-term developmental follow-up of children who are exposed to the pandemic has increased (3).

Early life experiences can affect the structure and connectivity of the developing infant brain (4), leading to potential delays in cognitive, language, and motor development during sensitive and vulnerable periods. A meta-analysis of neurodevelopmental outcomes during COVID-19 have shown that the COVID-19 environment itself has a negative impact on communication at first year of life (5). Moreover, a recent study in Japan found that children aged 1–3 years exposed to COVID-19 were on a normal developmental trajectory, while children aged 3–5 years exposed to the pandemic had a 4.39-month delay in overall development compared to the pandemic group (6).

Very low birth weight (VLBW) infants weighing <1,500 g at birth, with relatively immature brains, are at risk of the negative effects of the pandemic with the pre-existing vulnerabilities associated with prematurity-related factors (7). Previous meta-analyses of the intellectual quotient (IQ) scores of VLBW children and adults reported the IQ score differences of −0.86 SD and −0.78 between VLBW and term-born children and adults, respectively, which is equivalent to approximately 12–13 IQ points (8). A few (9–11) studies have explored the association between the COVID-19 pandemic and neurodevelopment status in infants and preschoolers, including preterm infants. Shufferey et al. (9) showed 317 infants, including 17 preterm infants, had delays in gross motor, fine motor, and personal-social ability at 6 months of age regardless of maternal COVID-19 infection. Giesbrecht et al. (10) identified pandemic-born infants who were born more than 33 weeks of gestational age (GA) had lower scored in gross motor, communication, and personal-social skills than pre-pandemic cohort at 1-year-old. David et al. (11) discovered 132 VLBW infants had experienced cognitive and language delay at 20 months of corrected age. Given the adverse influence of COVID-19 era on neurodevelopmental disorder in high-risk population, impact of COVID-19 era on neurodevelopmental function deserves special consideration in VLBW infants. Strict neuromonitoring and the provision of specific interventions that are beneficial for preterm children should be employed to achieve normal neurodevelopmental outcomes.

Our nationwide cohort, the Korean Neonatal Network (KNN), aimed to detect neurodevelopmental problems early through a follow-up program. Although the COVID-19 era may affect the neurodevelopmental outcomes of VLBW infants through a possible influence, studies exploring this aspect are limited. To understand the negative effects of the COVID-19 pandemic on neurodevelopment in VLBW infants who experienced the pandemic, we aimed to explore the association between exposure to COVID-19 and neurodevelopmental outcomes at 18–24 months and 33–39 months years using data from the KNN in a cross-sectional study.

## 2 Methods

### 2.1 Data source and study population

KNN is a web-based network targeting neonates born with a birth weight of less than 1,500 g or GA of less than 32 weeks from neonatal intensive care units (NICUs) nationwide, representing 70% of the NICU beds in the country. NICU follow-up is important to detect the developmental delay of preterm infants. The database contains prospectively collected clinical information of 70%–80% of VLBW infants born in Korea each year, using a standardized electronic case report form. NICU follow-up is important to detect the developmental delay of preterm infants. Currently, 79 hospitals are participating in the KNN, with a cohort count of 20,427.

**Figure 1:**
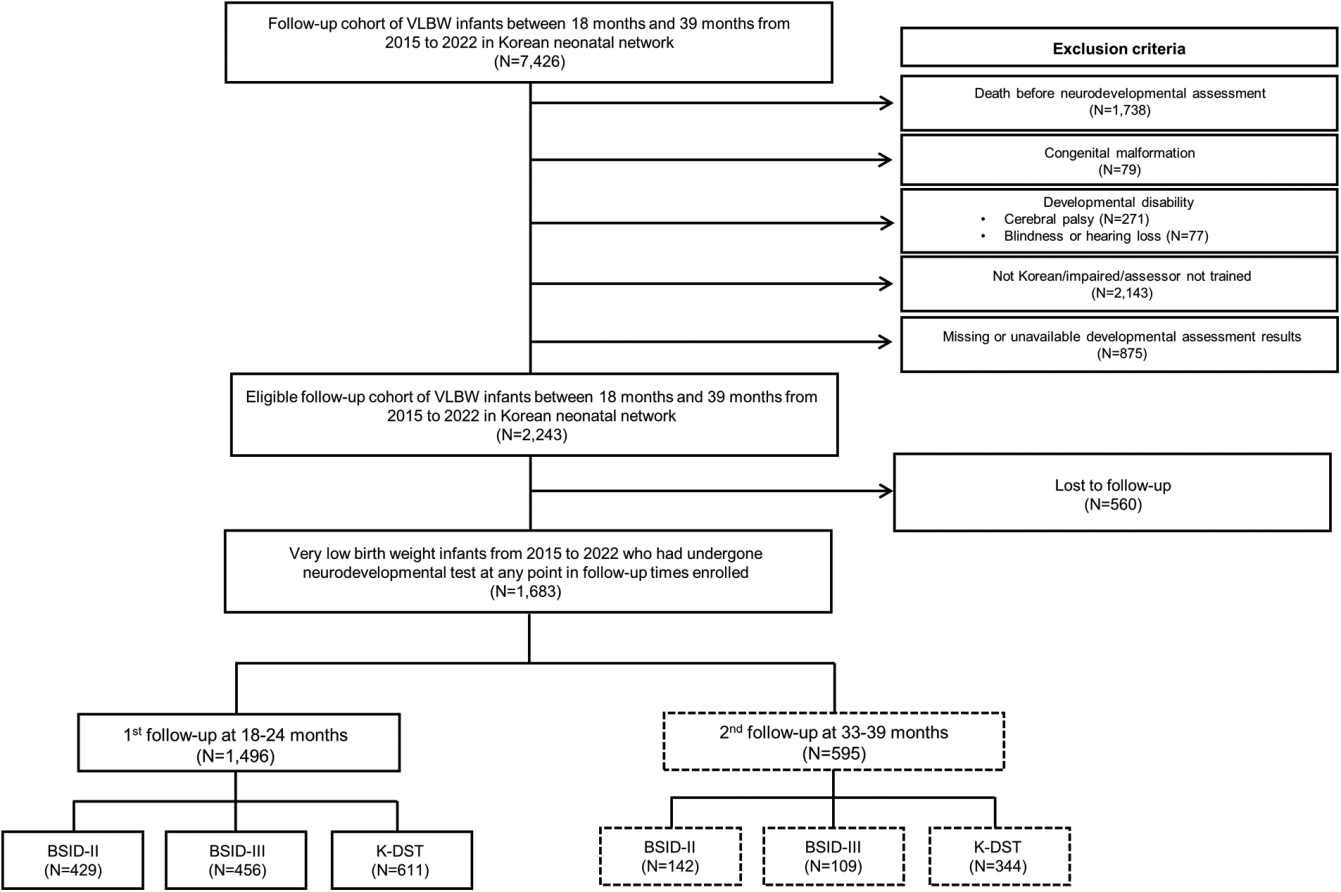
Flowchart showing the participant selection process. VLBW, very low birth weight; BSID, Bayley Scales of Infant Development; K-DST, Korean Developmental Screening Test for Infants and Children.

Data from 7,426 VLBW infants who were born and registered with the KNN between January 2015, and May 2022, were used in the study. We excluded VLBW infants who died before neurodevelopmental assessment (*N* = 1,738); were diagnosed with congenital malformation (*N* = 79), cerebral palsy (*N* = 271), blindness, or hearing loss (*N* = 77); not Korean/impaired/assessor not trained (*N* = 2,143); and missing or unavailable developmental assessment results (*N* = 875). Among the eligible follow-up cohorts (*N* = 2,243), those who were lost to developmental testing were excluded (*N* = 560). A total of 1,683 VLBW infants who underwent neurodevelopmental tests at 18–24 months and/or 33–39 months of age were enrolled. We analyzed the long-term neurodevelopmental outcomes using BSID and Korean developmental screening test for infants and children (K-DST) at corrected ages of 18–24 months (*N* = 1,496) and 33–39 months (*N* = 595). The characteristics of the study participants are shown in Figure 1.

To compare the demographics of infants who were lost to follow-up with those included in the analysis, we conducted propensity score matching (PSM), correcting for perinatal factors such as gestational age (GA), birth weight, and sex (*N* = 4,005). The cohort used in the analysis had lower rates of clinical mobility (bronchopulmonary dysplasia (BPD) ≥ moderate, intraventricular hemorrhage (IVH) ≥ grade III, periventricular leukomalacia (PVL) only) than infants who were lost to follow-up (Supplementary Table 1).

### 2.2 Socioeconomic status (SES)

To investigate the impact of environmental factors, specifically SES, on the neurodevelopment of VLBW infants, maternal education level and age were used. The maternal education was included as a primary measure of SES given prior study to use it as the most informative measures of SES in preterm birth at the time of NICU admission (12). To represent years of education, we applied the level of maternal education to quantitative variable. The primary school, secondary school, high school, and graduated college or university were substituted with 6, 9, 12, 14, respectively.

### 2.3 COVID-19 exposure

From 2020 to 2021, the Korean government introduced regulations and recommendations for practicing social distancing in daily life. In this period (from May 2020 to May 2021), there was an increased emphasis on implementing social distancing policies to improve both individual and collective infection control measures, with the aim of hindering the transmission of the virus. According to the social distancing polices in Korea, we divided the cohort into the pandemic group and the pre-pandemic group. The pandemic group consisted of those who experienced social distancing for at least 1 year and underwent neurodevelopmental tests from May 2021 to May 2022. The pre-pandemic group comprised those who were born and underwent neurodevelopmental assessments between January 2015, and December 2019, before the COVID-19 outbreak.

### 2.4 Neurodevelopmental outcomes

As part of the standard procedure, the KNN regularly conducted developmental tests using the BSID-II, BSID-III, and K-DST on preterm infants at two specific time points: 18–24 months (1st follow-up) and 33–39 months of age (2nd follow-up). Not all the assessments were conducted simultaneously. All assessments were conducted in person at the hospital, with visible face masks worn, and took approximately 30 min–1 h to complete.

BSID is a widely used assessment tool for measuring the mental, motor, and behavioral development of preterm infants from 1 to 42 months and is commonly implemented to monitor the neurodevelopmental outcomes (13). BSID-II was useful tool for high-risk infants consisted of a mental development index (MDI) and the other is psychomotor development index (PDI). Below −1SD (<84) in MDI or PDI was defined mental or motor development delay, and the severe motor development delay was defined as an MDI or PDI of <68 (below −2SD). With the revision of BSID to III, BSID-II is gradually being replaced by BSID-III in clinical practice. The BSID-III was comprised of three areas of development: cognitive, language, and motor, and two parent-report subtests reflecting social-emotional development. Scores below 85 (<-1SD) on the BSID-III are considered equivalent to being one standard deviation below the mean, while scores below 70 (<-2SD) are considered equivalent to being two standard deviations below the mean.

The K-DST was specifically developed and modified to detect potential developmental delays considering the cultural and newborn care environment in Korea. It is a standardized assessment with a sensitivity of 0.833 and specificity of 0.979 widely used in hospitals across Korea sponsored by the Ministry of Health and Welfare (14). The test demonstrated its validity by distinguishing between normal and neurodevelopmentally delayed groups (15). It consists of questions covering six domains: gross motor function, fine motor function, cognition, language, social interaction, and self- help (15). However, self-help ability was not measured in this study because it only emerges after a certain developmental stage. The total score for each domain was categorized into four levels: higher than peer-level (≥1SD), peer-level (≤1SD and >−1SD), follow-up evaluation is recommended (<−1SD and ≥−2SD), and detailed evaluation is warranted (<−2SD).

### 2.5 Graphical network analysis (GNA)

To analyze preterm infants robustly, a graphical approach that suggests potential outcomes should be used (16). GNA is a valuable statistical method for identifying complex relationships between variables that model the partial correlation structure of a set of variables (17) and has been used as a powerful tool to determine the conditional effects between neurodevelopmental outcomes and the clinical characteristics of preterm infants (18). Moreover, GNA aims to clarify complex relationships and identify key influences by representing them as a network of interconnected nodes and edges. The present study identified significant pairwise conditional correlations between neurodevelopmental assessment and clinical characteristics. With the risk factors identified using the GNA, to enhance the effect of COVID-19 exposure on neurodevelopmental outcomes more rigorously, we developed the adjusted fitting models based on GNA results. Moreover, generalized linear models were constructed using independent covariance structures for all predictors; then, the percentage variance (partial R^2^) of each predictor was extracted for each clinical outcome.

### 2.6 Adjusted model for neurodevelopmental outcomes

To identify the association between COVID-19 exposure and neurodevelopmental outcomes, multiple regression models were used. We developed the three adjusted fitting models that incorporated the clinical factors and stepwise regression approach. Variables related to the environmental factors (maternal age and level of education) and morbidity BPD ≥ moderate and IVH grade III or higher) were progressively included. First, Model 1 was adjusted only for perinatal factors. Model 2 was analyzed adjusting for perinatal factors and clinical mobility. Finally, to account for the independent of variance about the effects COVID-19 exposure on neurodevelopmental outcomes, we adjusted for all the associated factors such as preterm birth-related morbidities and environmental variables in Model 3.

### 2.7 Statistical analysis

Descriptive statistics were used to summarize the demographic and clinical characteristics of the study population. Continuous variables were expressed as the means and standard deviations, while categorical variables were expressed as frequencies and percentages. The differences between the pandemic and pre-pandemic groups were compared using a *t*-test for continuous variables and an *χ*^2^ test for categorical variables. The statistical threshold was set at a two-tailed *p* value of <0.05. Analyses were performed using SPSS version 28 (SPSS, Chicago, IL, USA) and R version 4.3.1 (R Foundation for Statistical Computing, Vienna, Austria).

## 3 Results

### 3.1 Clinical characteristics

Of the 1,683 VLBW infants who were assessed in any case 1 test in either of the two follow-up periods, 88.89% [total infants: 1,496; male infants: 773 (51.67%)] were evaluated at 18–24 months and 35.35% [total infants: 595; male infants: 298 (50.08%)] at 33–39 months. Among the 1,496 children assessed at the first follow-up, 1,267 were in the pre-pandemic group and 229 were in the pandemic group. At the second follow-up, the 595 children assessed included 479 in the pre-pandemic group and 116 in the pandemic group. The clinical characteristics of the patients included in this study are shown in Table 1. The demographics of the pre-pandemic group were compared with those of the pandemic group in Supplementary Tables 2–S4. The incidence of PDA ligation was higher in the pre-pandemic group, while that of BPD ≥moderate was higher in the pandemic group.

**Table 1:** Clinical characteristics of the study cohort.

	1st follow-up (*N* = 1,496)			2nd follow-up (*N* = 595)		
	Pre-pandemic (*N* = 1,267)	Pandemic (*N* = 229)	*p*	Pre-pandemic (*N* = 479)	Pandemic (*N* = 116)	*p*
Maternal characteristics						
Maternal age, years	33.43 ± 4.10	34.32 ± 3.69	**0**.**002**	33.33 ± 4.35	33.59 ± 3.52	0.542
Maternal education	13.57 ± 0.91 (*N* = 1,077)	13.67 ± 0.86 (*N* = 170)	0.182	13.55 ± 0.86 (*N* = 421)	13.67 ± 0.74 (*N* = 98)	0.193
High school	207/1,077 (19.2)	23/170 (13.5)	0.075	92/421 (21.9)	16/98 (16.3)	0.175
College or University	861/1,077 (79.9)	145/170 (85.3)	0.101	328/421 (77.9)	30/98 (83.7)	0.207
GDM	103/1,246 (8.3)	21/222 (9.5)	0.556	38/470 (8.1)	3/111 (2.7)	**0**.**046**
Preeclampsia	139/1,241 (11.2)	32/222 (14.4)	0.170	60/467 (12.8)	16/114 (14.0)	0.736
Histologic chorioamnionitis	601/1,129 (53.2)	104/220 (47.3)	0.105	236/431 (54.8)	51/111 (45.9)	0.097
Infant characteristics						
Gestational age, weeks	26.57 ± 1.38	26.27 ± 1.42	**0**.**002**	26.44 ± 1.44	26.55 ± 1.46	0.439
Birth weight, g	968.52 ± 224.46	908.69 ± 224.42	**<0**.**001**	951.92 ± 233.28	955.04 ± 242.89	0.898
Head circumference	24.70 ± 1.98 (*N* = 1,200)	24.15 ± 1.89 (*N* = 217)	**<0**.**001**	24.56 ± 1.95 (*N* = 455)	24.67 ± 2.27 (*N* = 105)	0.614
Cesarean section	960 (75.8)	177 (77.3)	0.619	363 (75.8)	93 (80.2)	0.316
Male sex	654 (51.6)	119 (52.0)	0.923	240 (50.1)	58 (50.0)	0.984
Apgar score at 1 min	4.23 ± 1.88 (*N* = 1,260)	4.35 ± 1.85	0.374	4.07 ± 1.86 (*N* = 476)	4.47 ± 2.09	0.061
Apgar score at 5 min	6.60 ± 1.77 (*N* = 1,260)	6.60 ± 1.82	0.984	6.51 ± 1.73 (*N* = 477)	6.74 ± 1.96	0.207
Antenatal corticosteroid	1,098/1,252 (87.7)	224 (97.8)	**<0**.**001**	411/474 (86.7)	105/115 (91.3)	0.180
Resuscitation	1,224/1,264 (96.8)	225/228 (98.7)	0.125	459/477 (96.2)	113/115 (98.3)	0.278
BPD ≥ moderate	557/1,264 (44.1)	121/227 (53.3)	**0**.**010**	215/478 (45.0)	61 (52.6)	0.141
Surfactant	1,192 (94.1)	222 (96.9)	0.080	451 (94.2)	108 (93.1)	0.670
ROP ≥ stage III	263/628 (41.9)	52/139 (37.4)	0.333	108/254 (42.5)	32/76 (42.1)	0.949
IVH ≥ grade III	74 (5.8)	22 (9.6)	**0**.**032**	26 (5.4)	14 (12.1)	**0**.**010**
PVL only	19 (1.5)	5 (2.2)	0.448	7 (1.5)	3 (2.6)	0.398
PDA ligation	234/792 (29.5)	41/108 (38.0)	0.075	82/317 (25.9)	25/47 (53.2)	**<0**.**001**
NEC ≥ stage2	109 (8.6)	15 (6.6)	0.300	34 (7.1)	12 (10.3)	0.240
PROM	542/1,258 (43.1)	103 (45.0)	0.595	216/477 (45.3)	37/115 (32.2)	**0**.**011**
Sepsis	346 (27.3)	54 (23.6)	0.241	132 (27.6)	27 (23.3)	0.350
RDS	1,185 (93.5)	220 (96.1)	0.139	442 (92.3)	110 (94.8)	0.341
TPN days	36.74 ± 28.30	37.67 ± 26.89	0.645	36.79 ± 26.71	40.67 ± 29.16	0.163

Data are expressed as the mean ± standard deviation or *n* (%). GDM, gestational diabetes mellitus; BPD, bronchopulmonary dysplasia; ROP, retinopathy of prematurity; IVH, intraventricular hemorrhage; PVL, periventricular leukomalacia; PDA, patent ductus arteriosus; NEC, necrotizing enterocolitis; PROM, premature rupture of membranes; RDS, respiratory distress syndrome; TPN, total parenteral nutrition. Significant group differences (*p* < 0.05) are highlighted in bold.

### 3.2 Neurodevelopmental outcomes between the two groups

In the 18–24-month follow-up cohort, the pandemic group scored significantly lower in the language (*p *= 0.026) and motor (*p *= 0.018) domains of the BSID-III than the pre-pandemic group. Additionally, a significantly higher proportion of patients scored less than −1SD in the language domain of the K-DST (*p *= 0.022). In the 3-year cohort, the pandemic group scored significantly lower in the cognitive (*p *= 0.026) and language (*p *= 0.041) domains of the BSID-III than the pre-pandemic group. Furthermore, the proportion of patients who scored less than −1SD was significantly higher in the gross motor domain of the K-DST (*p *= 0.008). In the BSID-II, there was no difference in the age at the time of assessment between the groups. While the assessment age was higher in both the BSID-III and the K-DST for the pandemic group, the developmental scores were lower in the pandemic group. The neurodevelopmental outcomes at both follow-up periods are shown in Table 2.

**Table 2:** Neurodevelopmental outcomes at 18–24 months and 33–39 months of the two study groups.

	Pre-pandemic	Pandemic	*p*
18–24 months			
BSID-II	(*N* = 388)	(*N* = 41)	
Age at assessment (months)	20.39 ± 2.40	21.12 ± 2.87	0.122
MDI	85.75 ± 19.10	84.46 ± 21.75	0.686
PDI	86.80 ± 17.31	84.93 ± 18.37	0.514
BSID-III	(*N* = 323)	(*N* = 133)	
Age at assessment (months)	19.29 ± 1.75	20.90 ± 2.14	<0.001
Cognitive	94.88 ± 14.83	92.26 ± 16.88	0.101
Language	89.62 ± 14.92	86.11 ± 15.89	**0**.**026**
Motor	95.31 ± 13.13	91.98 ± 14.68	**0**.**018**
K-DST (≤−1SD), *N* (%)	(*N* = 556)	(*N* = 55)	
Age at assessment (months)	19.48 ± 2.01	21.60 ± 2.32	<0.001
Gross motor	180 (32.4)	20 (36.4)	0.548
Fine motor	150 (27.0)	20 (36.4)	0.138
Cognition	168 (30.2)	22 (40.0)	0.135
Language	187 (33.6)	27 (49.1)	**0**.**022**
Sociality	128 (23.0)	17 (30.9)	0.190
33–39 months			
BSID-II	(*N* = 131)	(*N* = 11)	
Age at assessment (months)	35.47 ± 31.17	35.91 ± 2.39	0.651
MDI	88.28 ± 20.56	89.45 ± 16.75	0.854
PDI	78.34 ± 18.96	76.09 ± 20.11	0.707
BSID-III	(*N* = 47)	(*N* = 62)	
Age at assessment (months)	33.32 ± 3.58	36.35 ± 2.17	<0.001
Cognitive	90.26 ± 14.57	83.39 ± 17.14	**0**.**026**
Language	84.28 ± 14.50	77.47 ± 19.81	**0**.**041**
Motor	82.74 ± 13.69	82.73 ± 15.54	0.082
K-DST (≤−1SD), *N* (%)	(*N* = 301)	(*N* = 43)	
Age at assessment (months)	35.06 ± 1.41	36.00 ± 0.00	<0.001
Gross motor	53 (17.6)	15 (34.9)	**0**.**008**
Fine motor	82 (27.2)	11 (25.6)	0.819
Cognition	70 (23.3)	12 (27.9)	0.503
Language	103 (34.2)	12 (27.9)	0.412
Sociality	64 (21.3)	10 (23.3)	0.766

Data are expressed as the mean ± SD or N (%). SD, standard deviation; BSID, Bayley scale of infant development; MDI, mental developmental index; PDI, psychomotor developmental index; K-DST, Korean Developmental Screening Test for Infants and Children. Significant group differences (*p* < 0.05) are highlighted in bold.

### 3.3 Pairwise conditional effects determined by GNA at 18–24 months and 33–39 months of follow-up

**Figure 2:**
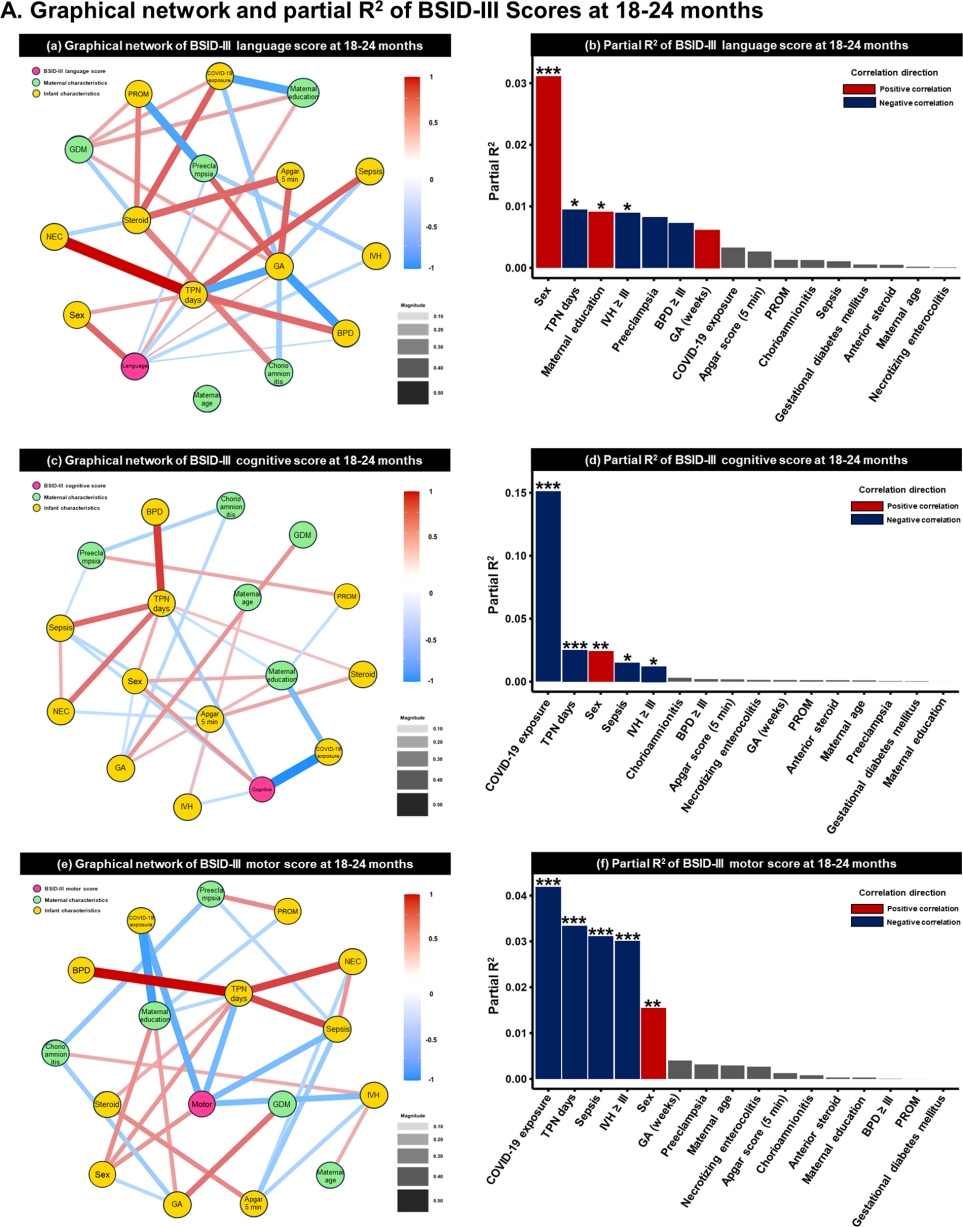
Graphical network analysis of BSID-III scores at **(A)** 18–24 months (pre-pandemic =323, pandemic = 133) and **(B)** 33–39 months (pre-pandemic = 47, pandemic = 62). **(a,c,e)** at 18–24 months and (**g,i,k)** display the correlation of each variable using a graphical network analysis. The magnitude of the line between variables indicates the strength of association, while the color indicates directionality. Red lines represent a positive association, while blue lines depict a negative association. **(b,d,f)** at 18–24 months and **(h,j,l)** at 33–39 months exhibit the partial R^2^, suggesting the coefficient of determination. BSID; Bayley Scales of Infant Development; GDM, gestational diabetes mellitus; BPD, bronchopulmonary dysplasia; IVH, intraventricular hemorrhage; NEC, necrotizing enterocolitis; PROM, premature rupture of membranes; TPN, total parenteral nutrition. *p < 0.05, **p < 0.01, and ***p < 0.05.

As a result of the GNA based on the BSID-III score, COVID-19 exposure had the largest effect on BSID-III cognition (1st follow-up: partial R^2 ^= 0.150, *p *< 0.001; 2nd follow-up: partial R^2 ^= 0.042, *p *= 0.047) and motor (1st follow-up: partial R^2 ^= 0.042, *p *< 0.001; 2nd follow-up: partial R^2 ^= 0.047, *p *= 0.036) scores at both time points. The next most influential factor was sex, with male infants scoring lower than female infants. SES was associated with language scores at 18 months (partial R^2 ^= 0.009; *p *= 0.049). As a single factor in the fully adjusted generalized linear model, the effect of total parenteral nutrition days decreased with age. Preterm birth-related factors such as IVH ≥grade III and sepsis had an effect at 1st follow-up but were offset at 2nd follow-up. The GNA results and partial R^2^ of each developmental domain are shown in Figure 2 and Supplementary Tables 5–S7.

**Figure:**
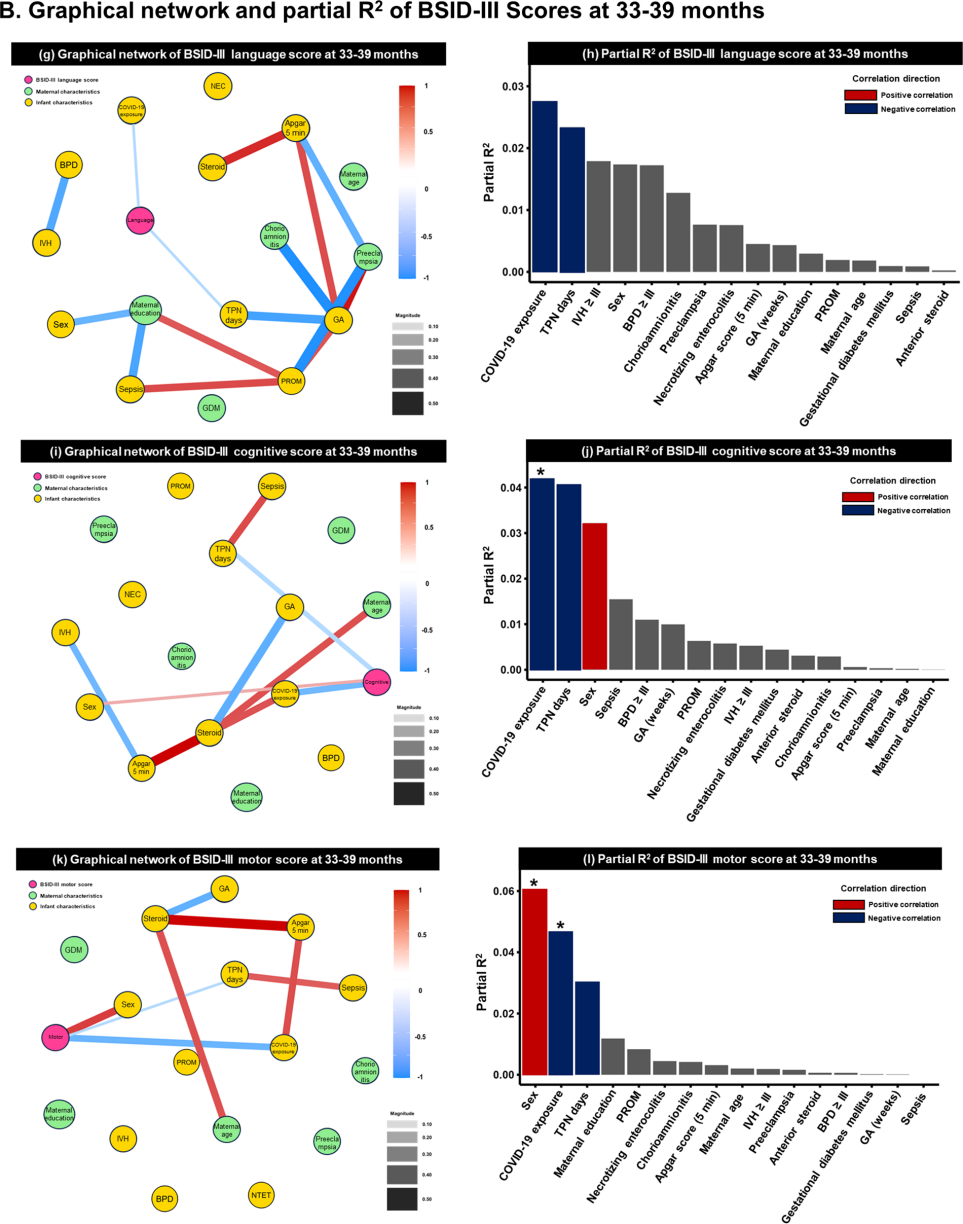


### 3.4 Adjusted models of neurodevelopmental outcomes

In the 18–24-month cohort, after adjusting for clinical morbidity, and/or environmental factors, the pandemic group scored significantly lower in the language domain of the BSID-III (model 3, B = −4.029, *p *= 0.021) and high proportion of ≤−1SD in language subdomains of the K-DST (model 3, B = 2.713, *p *= 0.007) than the pre-pandemic group. However, the association between COVID-19 exposure and motor outcomes was weakened in the multivariate analysis after adjusting for SES at 18–24 months of age (model 2, B = −2.735, *p *= 0.040; model 3, B = −1.923, *p *= 0.196). In the 3-year cohort, after adjusting for clinical morbidity, and/or environmental factors, the pandemic group scored significantly lower in the cognitive (model 1, B = −8.017, *p *= 0.011; model 2, B = −8.350, *p *= 0.008; model 3, B = −8.324, *p *= 0.013) and language (model 1, B = −7.909, *p *= 0.023; model 2, B = −8.312, *p *= 0.017; model 3, B = −8.437, *p *= 0.023) domains of the BSID-III than the pre-pandemic group. Furthermore, a significantly higher proportion of patients scored less than −1SD in the gross motor domain of the K-DST (model 1, B = 2.552, *p *= 0.009; model 2, B = 2.894, *p *= 0.005; model 3, B = 4.455, *p *< 0.001). The adjusted models are listed in Table 3.

**Table 3:** Adjusted models of neurodevelopmental outcomes according to the duration of COVID-19 exposure.

	Model 1^a^			Model 2^b^			Model 3^c^		
	B (unstandardized coefficients)	95% CI	*p*	B (unstandardized coefficients)	95% CI	*p*	B (unstandardized coefficients)	95% CI	*p*
18–24 months									
BSID-II	(*N* = 429)			(*N* = 428)			(*N* = 342)		
MDI	−1.615	−7.818–4.588	0.609	−0.584	−6.792–5.623	0.853	−1.454	−8.013–5.105	0.663
PDI	−2.080	−7.581–3.420	0.458	−1.109	−6.574–4.355	0.690	−1.067	−6.951–4.817	0.721
BSID-III	(*N* = 456)			(*N* = 454)			(*N* = 381)		
Cognitive	−1.802	−4.906–1.302	0.254	−2.009	−5.054–1.035	0.195	−0.806	−4.278–2.666	0.648
Language	−2.500	−5.518–0.519	0.104	−2.711	−5.687–0.266	0.074	−4.029	−7.443–−0.615	**0**.**021**
Motor	−2.346	−5.038–0.347	0.088	−2.735	−5.343 to −0.128	**0**.**040**	−1.923	−4.841–0.996	0.196
K-DST (≤−1SD)	(*N* = 611)			(*N* = 609)			(*N* = 519)		
Gross motor	1.114	0.614–2.019	0.723	0.868	0.460–1.640	0.663	1.345	0.651–2.779	0.423
Fine motor	1.493	0.822–2.712	0.188	1.287	0.688–2.407	0.429	1.466	0.710–3.024	0.301
Cognition	1.502	0.837–2.694	0.172	1.306	0.714–2.388	0.387	1.886	0.930–3.823	0.078
Language	1.879	1.059–3.335	**0**.**031**	1.630	0.903–2.944	0.105	2.713	1.319–5.583	**0**.**007**
Sociality	1.450	0.782–2.691	0.238	1.338	0.709–2.525	0.369	1.777	0.862–3.661	0.119
33–39 months									
BSID-II	(*N* = 142)			(*N* = 142)			(*N* = 114)		
MDI	−0.060	−12.636–12.515	0.992	0.912	−12.151–13.975	0.890	3.015	−10.515–16.544	0.660
PDI	−4.213	−15.649–7.222	0.468	−0.681	−12.348–10.986	0.908	2.986	−9.351–15.323	0.632
BSID-III	(*N* = 109)			(*N* = 109)			(*N* = 93)		
Cognitive	−8.017	−14.136 to −1.898	**0**.**011**	−8.350	−14.500 to −2.200	**0**.**008**	−8.324	−14.842 to −1.807	**0**.**013**
Language	−7.909	−14.703 to −1.114	**0**.**023**	−8.312	−15.126 to −1.499	**0**.**017**	−8.437	−15.679 to −1.195	**0**.**023**
Motor	−6.261	−11.810 to −0.712	**0**.**027**	−6.157	−11.775 to −0.538	**0**.**032**	−5.618	−11.691 to 0.456	0.069
K-DST (≤−1SD)	(*N* = 344)			(*N* = 343)			(*N* = 311)		
Gross motor	2.552	1.264–5.153	**0**.**009**	2.894	1.382–6.061	**0**.**005**	4.455	1.996–9.942	**<0**.**001**
Fine motor	0.912	0.432–1.927	0.809	0.926	0.429–1.999	0.845	0.987	0.428–2.273	0.975
Cognition	1.297	0.614–2.740	0.495	1.299	0.592–2.854	0.514	1.187	0.499–2.825	0.699
Language	0.737	0.361–1.507	0.403	0.768	0.371–1.593	0.479	0.772	0.351–1.697	0.519
Sociality	1.130	0.504–2.534	0.767	1.205	0.526–2.764	0.659	1.371	0.573–3.281	0.479

One-way analysis of variance (ANOVA) for the BSID and logistic regression for the K-DST were used. ANOVA, analysis of variance; CI, confidence interval; BSID, Bayley scale of infant development; MDI, mental developmental index; PDI, psychomotor developmental index; K-DST, Korean Developmental Screening Test for Infants and Children; SD, standard deviation; BPD, bronchopulmonary dysplasia; IVH, intraventricular hemorrhage; GA, gestational age; SES, socioeconomic status.

^a^ Model 1 shows COVID-19, clinical morbidity (moderate BPD and grade III IVH), and SES (maternal age and education) on neurodevelopmental outcome adjusted for the GA and sex.

^b^ Model 2 shows COVID-19 and SES (maternal age and education) on neurodevelopmental outcome adjusted for the GA, sex, and clinical morbidity (moderate BPD, and grade III IVH).

^c^ Model 3 shows pure COVID-19 effect on neurodevelopmental outcome adjusted for the GA, sex, clinical morbidity (moderate BPD and grade III IVH), and SES (maternal age and education). Significant group differences (*p* < 0.05) are highlighted in bold.

## 4 Discussion

This prospective study is the first to explore the impact of COVID-19 exposure on the neurodevelopmental outcomes of VLBW infants, elucidating the potential predictors at both 18–24 months and 33–39 months of age. The pandemic group had significantly lower language scores on the BSID-III at 1st and 2nd follow-up periods. Notably, the pandemic group obtained significantly lower scores in the cognitive domains and showed gross motor delay at 2nd follow-up time compared with the pre-pandemic group.

Although the COVID-19 pandemic has led to increased hygiene practices such as wearing of face masks and washing of hands (19), COVID-19 has also acted as a “double-edged sword” of interaction and connection on social skills. A systematic review and meta-analysis showed that 7% of infants who underwent neurodevelopmental screening during the COVID-19 pandemic experienced neurodevelopmental disability (5). Few studies have examined the association between the pandemic and the development of healthy infants and children. Healthy children exposed to COVID-19 showed reduced early learning abilities (20), reduced gross or fine motor and communication skills (21) or personal-social function (9), increased internalizing and externalizing symptoms (22), and delayed overall development (6).

Despite these early findings, the effects of exposure to the COVID-19 environment on the neurodevelopmental consequences of VLBW remain unclear (Supplementary Table 8). Neurodevelopmental challenges observed in infancy may result from factors such as genetic predisposition, perinatal inflammation, infections, dietary factors, socioeconomic influences, maternal health, as well as congenital abnormalities. Current advances in perinatal care have substantially decreased the neurodevelopmental disabilities of preterm infants during the critical period (23). Nevertheless, short- and long-term follow-up studies have reported various neurodevelopmental disorders, such as cognitive and language impairments and intelligence disability, in VLBW children (24, 25). Notably, in VLBW preterm infants, experiencing COVID-19 for more than a year was associated with a higher risk of impairment in cognitive, language, and gross motor functions at 2nd follow-up period, after controlling for sex, neonatal morbidities, and SES. Only one study has investigated the development of VLBW infants before and after the COVID pandemic using the BSID-III at a corrected age of 20 months, which showed the language and cognitive scores of −6.2 and −8.6 points, respectively (11). However, the study only included a small sample size, as the participants were recruited from a single follow-up clinic and had no information on the duration of COVID-19 exposure. Although preterm morbidities were considered to identify the effect of COVID-19 on neurodevelopmental outcomes, not all factors were stratified and controlled according to potential influence. Our study is noteworthy as it identified that exposure to the COVID-19 environment increased the incidence of neurodevelopmental impairment until 3 years of age; the present study included a relatively large sample and used the nationwide KNN data. Meanwhile, it was noted that BSID-III scores were higher compared to BSID-II scores in a previous study (26). While a greater portion of the pandemic cohort underwent evaluation with BSID-III compared to the pre-pandemic group, preterm children who experienced the COVID-19 pandemic demonstrated lower scores than those who did not experience it.

Although COVID-19 exposure had the most significant impact on cognitive and motor development, sex, preterm-related factors, and SES were also notable variables in language development at 18–24 months. When planning to conduct neurodevelopmental studies focused on investigating the effects of early life exposure to the COVID-19 pandemic in preterm infants, sex, neonatal morbidities, and SES should be considered important prognostic factors. Neurodevelopmental impairment was more pronounced in male infants than in female infants. These findings are in line with that of a previous study showing that VLBW male infants were at a high risk of motor, language, and cognitive developmental problems at 2 years of age (18). Social factors are linked to brain development during childhood (27), and SES is particularly related to neurodevelopment in preterm infants (28). After adjusting for SES, the impact of COVID-19 exposure on motor development diminished, indicating that motor development was more influenced by SES than by COVID-19 exposure. This finding corresponds well with that of a previous preterm study, in which a high level of maternal education was related to higher motor and language BSID-III scores (BSID-III 8.23 and 15.74 points, respectively) at the age of 2 years (29).

Three potential mechanisms can explain the language impairment in VLBW infants at 1st and 2nd follow-up periods during the COVID-19 era. First, based on brain developmental theory, the first 3 years of a child's life are commonly recognized as a critical and sensitive period for development, including language (30). Infants learn receptive and expressive language abilities by nature, exposing their linguistic environments, including speaking, hearing, and communicating with others (31). Therefore, VLBW infants exposed to a fragile environment owing to the COVID-19 outbreak at a developmentally critical time are likely to experience developmental setbacks. Second, in the COVID-19 era, wearing face masks, which reduce opportunities to hear the caregiver's voice and detect mouth shape, could hinder the pivotal development of socio-emotional communication (32) and language ability (33). As lip-reading plays a crucial role in language development in the first 3 years of age (34), wearing face masks may have a negative impact on the normative language milestones. In addition, because wearing face masks reduces in-person interactions with peers and non-family members, language development is not stimulated among VLBW infants. As observation of the mouth and lips by face-to-face interaction is an essential factor in language development (35), communicating while wearing a face mask can be an obstacle to language development. Third, from a neuropathological perspective, the microstructural deviations in brain development in preterm infants occur over a longer period, causing language problems and academic achievement (36). In particular, VLBW infants may have atypically developed microstructures of the corpus callosum and uncinate fasciculus, which are involved in oral language skills. Our results imply that VLBW infants who are at high risk of altered brain development related to language skills may experience aggravated linguistic sequelae during the COVID-19 pandemic.

In addition to the language domain, cognitive and motor dysfunctions occur with increasing age. Cognitive and language development are closely related and interdependent in that they both involve the acquisition and use of knowledge and skills (37). Language development provides the foundation for cognitive development as it allows children to acquire and use new information and ideas. The cognitive and language outcomes during the COVID-19 era in the present study are consistent with the findings of previous studies (33, 38). The MDI of Bayley-II encompassing the cognition and language domains, did not demonstrate significance, while significant differences were observed in the language domain of Bayley-III at 18–24 months. Similarly, significant differences were found in the cognition domain of Bayley-III, but not the MDI of Bayley-II at the age of 3. There seems to be limited finding of group difference across ages and domains assessed due to the conflation of cognition and language domain in MDI. In addition, the pandemic has caused inactivity in children, likely due to a significant decrease in physical activity. Motor dysfunction may be related to social restrictions and lifestyle changes during an extended pandemic period. Owing to the implementation of social distancing policies during the COVID-19 pandemic, the prevalence of engaging in sedentary behavior has increased, but the performance of outdoor activities has decreased (39). These policies limited opportunities for children to engage in physical activity routines and outdoor playtime (40, 41), as parents and children were required to stay home the entire day. A recent study investigated the incidence of gross motor dysfunction in healthy infants within the first 1 year of life during the COVID-19 era (9, 10) and found a close relationship between negative social responses and physical aspects. Our results support the findings of previous studies (42), which reported that social distancing policies during the COVID-19 pandemic may have negatively impacted the VLBW infants’ gross motor development, emphasizing the need for further research in this area.

## 5 Conclusion

Based on the findings of this study, exposure to COVID-19 is associated with a higher risk of neurodevelopmental dysfunction in VLBW infants. The pandemic group exhibited lower language scores at 1st and 2nd follow-up periods. Moreover, at 2nd follow-up period, the pandemic group showed significantly lower scores in the cognitive domains, with a mean difference of 7 points, and a significantly higher percentage of gross motor delay compared with the pre-pandemic group. These findings highlight the detrimental effects of COVID-19 on the neurodevelopment of VLBW infants. Therefore, targeted interventions and support should be provided to mitigate the impact of COVID-19 on the developmental outcomes of vulnerable infants.

## 6 Limitation

Some limitations must be acknowledged. First, we were unable to assess the prevalence of COVID-19 or perform antibody testing to confirm previous infection status. Second, the 2nd follow-up cohort comprised a relatively small sample size compared with the 1st follow-up cohort. Third, we could not measure the levels of sibling and maternal psychological distress. As the pandemic is still ongoing, previous studies examining children's development during the pandemic have not set a precise duration of exposure to social distancing policies. Hence, further research is needed to fully understand the impact of shorter or longer exposures and to explore the neurodevelopmental outcomes of high-risk populations, such as VLBW infants.

## Data Availability

The datasets presented in this article are not readily available because the datasets of the present study are governed by the KNN∼. Data Management Committee. Requests to access the datasets should be directed to Hyun Ju Lee, blesslee77@hanmail.net.
