# Supplementary material for: Impact of COVID−19 pandemic on neurodevelopmental outcome in very low birth weight infants: a nationwide cohort study
Source: Front Pediatr. 2024 Sep 5;12:1368677. doi: 10.3389/fped.2024.1368677 (PMC11410593; doi:10.3389/fped.2024.1368677)
Supplement: Supplementary file 1 [file Table1.docx]

**Supplementary table 1**. Clinical characteristics of infants who were lost to follow-up with those included in follow-up cohort and propensity score matching on sex, gestational age, and birth weight

|  | **Before PSM** | | | **After PSM** | |
| --- | --- | --- | --- | --- | --- |
|  | **Cohort**  **(N=1,683)** | **Non-cohort**  **(N=4,005)** | ***p*** | **Non-cohort**  **(N=1,683)** | ***p*** |
| **Maternal characteristics** |  |  |  |  |  |
| Maternal age, years | 33.55±4.43 | 33.54±4.07 | 0.957 | 33.48±4.46 | 0.686 |
| Maternal education | 14.37±1.29 (N=1402) | 14.21±1.50 (N=2714) | **<0.001** | 14.20±1.46 | **<0.001** |
| High school | 268 (19.1) | 599 (22.1) | **0.028** | 260 (22.9) | **0.019** |
| College or University | 1,122 (80.0) | 2,062 (76.0) | **0.003** | 853 (75.2) | **0.004** |
| GDM | 134 (8.0) | 396 (9.9) | 0.072 | 174 (10.3) | 0.054 |
| Preeclampsia | 197 (11.7) | 508 (12.7) | 0.192 | 224 (13.3) | 0.103 |
| Histologic chorioamnionitis | 778 (46.2) | 1,487 (37.1) | **<0.001** | 596 (35.4) | **<0.001** |
| **Infant characteristics** |  |  |  |  |  |
| Gestational age, weeks | 26.53±1.39 | 26.40±1.51 | **0.003** | 26.54±1.40 | 0.748 |
| Birth weight, g | 959.73±227.51 | 945.51±230.04 | **0.033** | 965.32±227.26 | 0.475 |
| Head circumference | 24.62±1.98 (N=1,590) | 24.54±2.01 (N=3,704) | 0.140 | 24.67±1.94 (N=1,591) | 0.496 |
| Cesarean section | 1,285 (76.4) | 3,136 (78.3) | 0.107 | 1,316 (78.2) | 0.202 |
| Male sex | 817 (48.5) | 1,893 (47.3) | 0.554 | 781 (46.4) | 0.286 |
| Apgar score at 1 min | 4.23±1.89 (N=1675) | 4.15±1.90 (N=3,982) | 0.135 | 4.19±1.87 (N=1,676) | 0.501 |
| Apgar score at 5 min | 6.59±1.78 (N=1676) | 6.49±1.76 (N=3,984) | **0.045** | 6.53±1.75 (N=1,678) | 0.295 |
| Antenatal corticosteroid | 1,485 (88.2) | 3,484 (87.0) | 0.401 | 1,468 (87.2) | 0.668 |
| Resuscitation | 1,631 (96.9) | 3,884 (97.0) | 0.171 | 1,636 (97.2) | 0.349 |
| BPD≥moderate | 775/1,678 (46.2) | 2,078/3,899 (53.3) | **<0.001** | 864/1,639 (52.7) | **<0.001** |
| Surfactant | 213/1,611 (13.2) | 533/3,664 (14.5) | 0.203 | 211/1,544 (14.3) | 0.373 |
| ROP≥stage III | 362/1,679 (21.6) | 938/3,959 (23.7) | 0.082 | 375/1,668 (22.5) | 0.520 |
| IVH≥grade III | 114 (6.8) | 468/3,998 (11.7) | **<0.001** | 181/1,680 (10.8) | **<0.001** |
| PVL only | 17 (1.0) | 122 (3.0) | **<0.001** | 56 (3.3) | **<0.001** |
| PDA ligation | 310/1,006 (30.8) | 694/2,296 (30.2) | 0.159 | 288/985 (29.2) | 0.303 |
| NEC≥stage2 | 137 (8.1) | 361 (9.0) | 0.459 | 136 (8.1) | 0.950 |
| PROM | 719 (42.7) | 1616 (40.3) | 0.197 | 683 (40.6) | 0.391 |
| Sepsis | 444 (26.4) | 1087 (27.1) | 0.679 | 409 (24.3) | 0.235 |
| RDS | 1,582 (94.0) | 3,818 (95.3) | **0.036** | 1,615 (96.0) | **0.009** |
| TPN days | 37.14±28.40 | 40.11±32.42 | **0.001** | 38.03±29.70 | 0.373 |

Data are expressed as the mean±standard deviation or n (%). PSM, propensity score matching; GDM, gestational diabetes mellitus; BPD, bronchopulmonary dysplasia; ROP, retinopathy of prematurity; IVH, intraventricular hemorrhage; PVL, periventricular leukomalacia; PDA, patent ductus arteriosus; NEC, necrotizing enterocolitis; PROM, premature rupture of membranes; RDS, respiratory distress syndrome; TPN, total parenteral nutrition

**Supplementary table 2**. Clinical characteristics of BSID-II cohort at both follow-up periods

|  | **BSID-II cohort** | | | | | |
| --- | --- | --- | --- | --- | --- | --- |
|  | **1^st^ follow-up (18-24 months)** | | | **2^nd^ follow-up (33–39 months)** | | |
|  | **Pre-pandemic**  **(N=388)** | **Pandemic**  **(N=41)** | ***p*** | **Pre-pandemic**  **(N=131)** | **Pandemic**  **(N=11)** | ***p*** |
| **Maternal characteristics** |  |  |  |  |  |  |
| Maternal age, years | 33.60±3.97 | 34.12±3.81 | 0.420 | 33.15±4.09 | 33.64±3.53 | 0.704 |
| Maternal education | 13.53±0.90 (N=305) | 13.68±0.74 (N=38) | 0.327 | 13.52±0.86 (N=104) | 13.80±0.63 (N=10) | 0.219 |
| High school | 66 (21.6) | 6 (15.8) | 0.404 | 25 (24.0) | 1 (10.0) | 0.312 |
| College or University | 237 (77.7) | 32 (84.2) | 0.358 | 79 (76.0) | 9 (90.0) | 0.312 |
| GDM | 25 (6.5) | 4 (10.0) | 0.408 | 9 (6.9) | 0 (0.0) | 0.390 |
| Preeclampsia | 36 (9.7) | 6 (15.4) | 0.260 | 16 (12.8) | 1 (9.1) | 0.721 |
| Histologic chorioamnionitis | 166 (48.5) | 21 (52.5) | 0.635 | 55 (44.7) | 7 (63.6) | 0.228 |
| **Infant characteristics** |  |  |  |  |  |  |
| Gestational age, weeks | 26.52±1.39 | 26.41±1.12 | 0.585 | 26.12±1.53 | 26.73±1.56 | 0.210 |
| Birth weight, g | 960.47±233.29 | 877.34±235.29 | **0.031** | 909.66±241.89 | 1018.18±180.55 | 0.149 |
| Head circumference | 24.35±2.09 | 24.73±1.73 | 0.627 | 24.21±2.02 (N=125) | 25.21±1.63 | 0.113 |
| Cesarean section | 308 (79.4) | 33 (80.5) | 0.867 | 107 (81.7) | 8 (72.7) | 0.467 |
| Male sex | 167 (43.0) | 24 (58.5) | 0.058 | 61 (46.6) | 5 (45.5) | 0.943 |
| Apgar score at 1 min | 4.47±1.82 | 4.73±1.43 | 0.283 | 4.78±1.68 | 4.45±1.81 | 0.541 |
| Apgar score at 5 min | 6.65±1.78 | 6.85±1.42 | 0.483 | 6.92±1.56 | 6.45±1.57 | 0.341 |
| Antenatal corticosteroid | 352 (91.0) | 40 (97.6) | 0.147 | 118 (90.1) | 11 (100.0) | 0.273 |
| Resuscitation | 376 (96.9) | 39 (95.1) | 0.541 | 126 (96.2) | 10 (90.9) | 0.404 |
| BPD≥moderate | 172 (44.4) | 25 (61.0) | **0.043** | 58 (44.3) | 8 (72.7) | 0.069 |
| Surfactant | 361 (93.0) | 41 (100.0) | 0.081 | 125 (95.4) | 11 (100.0) | 0.468 |
| ROP≥stage III | 85 (42.1) | 8 (42.1) | 0.998 | 33 (42.3) | 4 (66.7) | 0.247 |
| IVH≥grade III | 17 (4.4) | 4 (9.8) | 0.129 | 7 (5.3) | 2 (18.2) | 0.093 |
| PVL only | 7 (1.8) | 1 (2.4) | 0.775 | 3 (2.3) | 0 (0.0) | 0.612 |
| PDA ligation | 66 (30.0) | 9 (50.0) | 0.079 | 16 (21.1) | 2 (66.7) | 0.065 |
| NEC≥stage2 | 35 (9.0) | 3 (7.3) | 0.715 | 16 (12.2) | 0 (0.0) | 0.219 |
| PROM | 161 (61.6) | 20 (48.8) | 0.376 | 54 (41.5) | 5 (45.5) | 0.800 |
| Sepsis | 91 (23.5) | 16 (39.0) | **0.028** | 34 (26.0) | 5 (45.5) | 0.164 |
| RDS | 358 (92.3) | 40 (97.6) | 0.213 | 120 (91.6) | 11 (100.0) | 0.317 |
| TPN days | 38.06±30.55 | 36.56±19.60 | 0.759 | 40.18±29.96 | 41.00±39.05 | 0.932 |

Data are expressed as the mean±standard deviation or n (%). BSID, Bayley scales of infant and toddler development-III; GDM, gestational diabetes mellitus; BPD, bronchopulmonary dysplasia; ROP, retinopathy of prematurity; IVH, intraventricular hemorrhage; PVL, periventricular leukomalacia; PDA, patent ductus arteriosus; NEC, necrotizing enterocolitis; PROM, premature rupture of membranes; RDS, respiratory distress syndrome; TPN, total parenteral nutrition

**Supplementary table 3**. Clinical characteristics of BSID-III cohort at both follow-up periods

|  | **BSID-III cohort** | | | | | |
| --- | --- | --- | --- | --- | --- | --- |
|  | **1^st^ follow-up (18-24 months)** | | | **2^nd^ follow-up (33–39 months)** | | |
|  | **Pre-pandemic**  **(N=323)** | **Pandemic**  **(N=133)** | ***p*** | **Pre-pandemic**  **(N=47)** | **Pandemic**  **(N=62)** | ***p*** |
| **Maternal characteristics** |  |  |  |  |  |  |
| Maternal age, years | 33.78±4.03 | 34.11±3.37 | 0.418 | 33.60±5.14 | 33.73±3.82 | 0.880 |
| Maternal education | 13.61±0.89 (N=290) | 13.68±0.93 (N=93) | 0.554 | 13.60±0.81 (N=40) | 13.62±0.79 (N=53) | 0.893 |
| High school | 52 (17.9) | 10 (10.8) | 0.102 | 8 (20.0) | 10 (18.9) | 0.891 |
| College or University | 237 (81.7) | 81 (87.1) | 0.230 | 32 (80.0) | 43 (81.1) | 0.891 |
| GDM | 14 (4.4) | 10 (7.7) | 0.168 | 4 (8.5) | 2 (3.3) | 0.239 |
| Preeclampsia | 40 (12.5) | 18 (14.1) | 0.665 | 6 (13.0) | 13 (21.7) | 0.251 |
| Histologic chorioamnionitis | 149 (52.7) | 56 (44.4) | 0.125 | 18 (47.4) | 25 (41.7) | 0.579 |
| **Infant characteristics** |  |  |  |  |  |  |
| Gestational age, weeks | 26.55±1.43 | 26.18±1.48 | **0.012** | 26.04±1.63 | 26.45±1.48 | 0.174 |
| Birth weight, g | 957.81±218.45 | 901.08±212.20 | **0.011** | 864.38±215.84 | 913.82±252.52 | 0.284 |
| Head circumference | 24.66±2.05 (N=304) | 24.07±1.86 (N=126) | **0.006** | 24.04±1.74 (N=40) | 24.48±2.65 (N=55) | 0.363 |
| Cesarean section | 239 (74.0) | 98 (73.7) | 0.945 | 39 (83.0) | 52 (83.9) | 0.901 |
| Male sex | 156 (48.3) | 56 (42.1) | 0.228 | 20 (42.6) | 31 (50.0) | 0.440 |
| Apgar score at 1 min | 3.84±1.90 (N=321) | 4.43±1.87 | **0.003** | 2.87±1.72 | 4.27±2.14 | **<0.001** |
| Apgar score at 5 min | 6.36±1.74 (N=321) | 6.76±1.75 | **0.026** | 5.77±1.95 | 6.68±2.06 | **0.021** |
| Antenatal corticosteroid | 278 (87.7) | 131 (98.5) | **<0.001** | 40 (87.0) | 53 (85.5) | 0.827 |
| Resuscitation | 312 (96.9) | 132 (100.0) | **0.041** | 46 (97.9) | 61 (100.0) | 0.252 |
| BPD≥moderate | 155 (48.1) | 65 (49.2) | 0.830 | 28 (59.6) | 39 (62.9) | 0.724 |
| Surfactant | 300 (92.9) | 127 (95.5) | 0.299 | 46 (97.9) | 58 (93.5) | 0.285 |
| ROP≥stage III | 58 (36.3) | 31 (34.8) | 0.823 | 10 (35.7) | 23 (46.9) | 0.338 |
| IVH≥grade III | 23 (7.1) | 8 (6.0) | 0.670 | 4 (8.5) | 7 (11.3) | 0.633 |
| PVL only | 7 (2.2) | 2 (1.5) | 0.643 | 3 (6.4) | 2 (3.2) | 0.435 |
| PDA ligation | 69 (31.4) | 18 (29.5) | 0.782 | 13 (38.2) | 11 (50.0) | 0.385 |
| NEC≥stage2 | 27 (8.4) | 9 (6.8) | 0.567 | 3 (6.4) | 7 (11.3) | 0.379 |
| PROM | 142 (44.5) | 61 (45.9) | 0.793 | 17 (36.2) | 20 (32.8) | 0.713 |
| Sepsis | 103 (31.9) | 34 (25.6) | 0.181 | 20 (42.6) | 15 (24.2) | **0.042** |
| RDS | 302 (93.5) | 127 (95.5) | 0.413 | 46 (97.9) | 58 (93.5) | 0.285 |
| TPN days | 36.00±28.13 | 38.22±28.98 | 0.448 | 46.70±26.98 | 47.03±29.14 | 0.952 |

Data are expressed as the mean±standard deviation or n (%). BSID, Bayley scales of infant and toddler development-III; GDM, gestational diabetes mellitus; BPD, bronchopulmonary dysplasia; ROP, retinopathy of prematurity; IVH, intraventricular hemorrhage; PVL, periventricular leukomalacia; PDA, patent ductus arteriosus; NEC, necrotizing enterocolitis; PROM, premature rupture of membranes; RDS, respiratory distress syndrome; TPN, total parenteral nutrition

**Supplementary table 4**. Clinical characteristics of K-DST cohort at both follow-up periods

|  | **K-DST cohort** | | | | | |
| --- | --- | --- | --- | --- | --- | --- |
|  | **1^st^ follow-up (18-24 months)** | | | **2^nd^ follow-up (33–39 months)** | | |
|  | **Pre-pandemic**  **(N=556)** | **Pandemic**  **(N=55)** | ***p*** | **Pre-pandemic**  **(N=301)** | **Pandemic**  **(N=43)** | ***p*** |
| **Maternal characteristics** |  |  |  |  |  |  |
| Maternal age, years | 33.10±4.21 | 34.98±4.31 | **0.002** | 33.37±4.34 | 31.42±24.18 | 0.965 |
| Maternal education | 13.57±0.93 (N=482) | 13.64±0.78 (N=39) | 0.636 | 13.56±0.86 (N=277) | 13.71±0.71 (N=35) | 0.232 |
| High school | 89 (18.5) | 7 (17.9) | 0.936 | 59 (21.3) | 5 (14.3) | 0.333 |
| College or University | 387 (80.3) | 32 (82.1) | 0.790 | 217 (78.3) | 30 (85.7) | 0.311 |
| GDM | 64 (11.7) | 7 (13.5) | 0.704 | 25 (8.5) | 1 (2.5) | 0.182 |
| Preeclampsia | 63 (11.5) | 8 (14.5) | 0.500 | 38 (12.8) | 2 (4.7) | 0.120 |
| Histologic chorioamnionitis | 286 (56.7) | 27 (50.0) | 0.342 | 163 (60.4) | 19 (47.5) | 0.123 |
| **Infant characteristics** |  |  |  |  |  |  |
| Gestational age, weeks | 26.63±1.35 | 26.36±1.47 | 0.174 | 26.63±1.33 | 26.65±1.43 | 0.939 |
| Birth weight, g | 980.36±221.43 | 950.47±242.69 | 0.344 | 983.98±226.27 | 998.33±235.99 | 0.699 |
| Head circumference | 24.82±1.95 (N=521) | 24.24±2.10 (N=50) | **0.047** | 24.78±1.92 (N=290) | 24.79±1.82 (N=39) | 0.977 |
| Cesarean section | 413 (74.3) | 46 (83.6) | 0.126 | 217 (72.1) | 33 (76.7) | 0.522 |
| Male sex | 290 (52.2) | 30 (54.5) | 0.735 | 158 (52.5) | 22 (51.2) | 0.870 |
| Apgar score at 1 min | 4.29±1.88 (N=551) | 3.87±1.98 | 0.121 | 3.95±1.85 (N=298) | 4.77±2.09 | **0.008** |
| Apgar score at 5 min | 6.71±1.77 (N=551) | 6.02±2.11 | **0.007** | 6.44±1.71 (N=299) | 6.91±1.91 | 0.104 |
| Antenatal corticosteroid | 468 (85.4) | 53 (96.4) | **0.024** | 253 (85.2) | 41 (97.6) | **0.026** |
| Resuscitation | 536 (96.8) | 54 (98.2) | 0.560 | 287 (96.0) | 42 (97.7) | 0.588 |
| BPD≥moderate | 230 (41.4) | 31 (57.4) | **0.024** | 129 (43.0) | 14 (32.6) | 0.194 |
| Surfactant | 531 (95.5) | 54 (98.2) | 0.348 | 280 (93.0) | 39 (90.7) | 0.583 |
| ROP≥stage III | 120 (45.1) | 13 (41.9) | 0.736 | 65 (43.9) | 5 (23.8) | 0.080 |
| IVH≥grade III | 255 (46.7) | 31 (60.8) | 0.054 | 15 (5.0) | 5 (11.6) | 0.082 |
| PVL only | 5 (0.9) | 2 (3.6) | 0.069 | 1 (0.3) | 1 (2.3) | 0.108 |
| PDA ligation | 99 (28.1) | 14 (48.3) | **0.022** | 53 (25.6) | 12 (54.5) | **0.004** |
| NEC≥stage2 | 47 (8.5) | 3 (5.5) | 0.439 | 15 (5.0) | 5 (11.6) | 0.082 |
| PROM | 239 (43.3) | 22 (40.0) | 0.638 | 145 (48.3) | 12 (27.9) | **0.012** |
| Sepsis | 152 (27.3) | 4 (7.3) | **0.001** | 78 (25.9) | 7 (16.3) | 0.171 |
| RDS | 525 (94.4) | 53 (96.4) | 0.544 | 276 (91.7) | 41 (95.3) | 0.405 |
| TPN days | 36.25±26.74 | 37.16±26.71 | 0.809 | 33.77±23.87 | 31.42±24.18 | 0.547 |

Data are expressed as the mean±standard deviation or n (%). K-DST, Korean developmental screening test for infants and children; GDM, gestational diabetes mellitus; BPD, bronchopulmonary dysplasia; ROP, retinopathy of prematurity; IVH, intraventricular hemorrhage; PVL, periventricular leukomalacia; PDA, patent ductus arteriosus; NEC, necrotizing enterocolitis; PROM, premature rupture of membranes; RDS, respiratory distress syndrome; TPN, total parenteral nutrition

**Supplementary table 5.** Partial R^2^ of BSID-III language score at both follow-up periods

|  | **1^st^ follow-up**  **18-24 months**  **(N=456)** | | | **2^nd^ follow-up**  **33–39 months**  **(N=109)** | | |
| --- | --- | --- | --- | --- | --- | --- |
|  | **partial R^2^** | ***p*** | ***FDR p*** | **partial R^2^** | ***p*** | ***FDR p*** |
| **Maternal characteristics** |  |  |  |  |  |  |
| Mother’s age, years | 0.000 | 0.816 | 0.816 | 0.002 | 0.682 | 0.768 |
| Mother’s education | 0.009 | **0.049** | 0.188 | 0.003 | 0.603 | 0.768 |
| GDM | 0.001 | 0.644 | 0.805 | 0.001 | 0.768 | 0.768 |
| Preeclampsia | 0.008 | 0.075 | 0.188 | 0.008 | 0.402 | 0.768 |
| Histologic chorioamnionitis | 0.001 | 0.512 | 0.805 | 0.013 | 0.278 | 0.768 |
| **Infant characteristics** |  |  |  |  |  |  |
| Gestational age, weeks | 0.006 | 0.094 | 0.207 | 0.004 | 0.529 | 0.831 |
| Male sex | 0.031 | **<0.001** | **<0.001** | 0.017 | 0.206 | 0.567 |
| Apgar 5 min | 0.003 | 0.267 | 0.420 | 0.005 | 0.520 | 0.831 |
| Antenatal steroid | 0.001 | 0.508 | 0.559 | 0.000 | 0.882 | 0.882 |
| BPD≥moderate | 0.007 | 0.073 | 0.201 | 0.017 | 0.882 | 0.882 |
| IVH≥grade III | 0.009 | **0.047** | 0.176 | 0.018 | 0.199 | 0.567 |
| NEC≥stage 2 | 0.000 | 0.837 | 0.837 | 0.008 | 0.405 | 0.831 |
| PROM | 0.001 | 0.438 | 0.559 | 0.002 | 0.674 | 0.882 |
| Sepsis | 0.001 | 0.508 | 0.559 | 0.001 | 0.775 | 0.882 |
| TPN days | 0.009 | **0.048** | 0.176 | 0.023 | 0.141 | 0.567 |
| COVID-19 exposure | 0.003 | 0.224 | 0.411 | 0.028 | 0.110 | 0.567 |

GDM, gestational diabetes mellitus; BPD, bronchopulmonary dysplasia; IVH, intraventricular hemorrhage; NEC, necrotizing enterocolitis; PROM, premature rupture of membranes; TPN, total parenteral nutrition

**Supplementary table 6.** Partial R^2^ of BSID-III cognition score at both follow-up periods

|  | **1^st^ follow-up**  **18-24 months**  **(N=456)** | | | **2^nd^ follow-up**  **33–39 months**  **(N=109)** | | |
| --- | --- | --- | --- | --- | --- | --- |
|  | **partial R^2^** | ***p*** | ***FDR p*** | **partial R^2^** | ***p*** | ***FDR p*** |
| **Maternal characteristics** |  |  |  |  |  |  |
| Mother’s age, years | 0.003 | 0.504 | 0.850 | 0.002 | 0.895 | 0.947 |
| Mother’s education | 0.000 | 0.929 | 0.929 | 0.012 | 0.947 | 0.947 |
| GDM | 0.000 | 0.680 | 0.850 | 0.000 | 0.525 | 0.947 |
| Preeclampsia | 0.003 | 0.657 | 0.850 | 0.002 | 0.861 | 0.947 |
| Histologic chorioamnionitis | 0.001 | 0.247 | 0.850 | 0.004 | 0.606 | 0.947 |
| **Infant characteristics** |  |  |  |  |  |  |
| Gestational age, weeks | 0.004 | 0.452 | 0.482 | 0.000 | 0.339 | 0.595 |
| Male sex | 0.015 | **0.001** | **0.004** | 0.060 | 0.084 | 0.308 |
| Apgar 5 min | 0.001 | 0.370 | 0.482 | 0.003 | 0.815 | 0.815 |
| Antenatal steroid | 0.000 | 0.482 | 0.482 | 0.001 | 0.595 | 0.645 |
| BPD≥moderate | 0.000 | 0.344 | 0.482 | 0.001 | 0.315 | 0.595 |
| IVH≥grade III | 0.030 | **0.023** | 0.506 | 0.002 | 0.487 | 0.595 |
| NEC≥stage 2 | 0.003 | 0.438 | 0.482 | 0.004 | 0.467 | 0.595 |
| PROM | 0.000 | 0.470 | 0.482 | 0.008 | 0.446 | 0.595 |
| Sepsis | 0.031 | **0.011** | **0.030** | 0.000 | 0.232 | 0.595 |
| TPN days | 0.033 | **<0.001** | **<0.001** | 0.030 | 0.051 | 0.281 |
| COVID-19 exposure | 0.151 | **<0.001** | **<0.001** | 0.042 | **0.047** | 0.281 |

GDM, gestational diabetes mellitus; BPD, bronchopulmonary dysplasia; IVH, intraventricular hemorrhage; NEC, necrotizing enterocolitis; PROM, premature rupture of membranes; TPN, total parenteral nutrition

**Supplementary table 7.** Partial R^2^ of BSID-III motor score at both follow-up periods

|  | **1^st^ follow-up**  **18-24 months**  **(N=456)** | | | **2^nd^ follow-up**  **33–39 months**  **(N=109)** | | |
| --- | --- | --- | --- | --- | --- | --- |
|  | **partial R^2^** | ***p*** | ***FDR p*** | **partial R^2^** | ***p*** | ***FDR p*** |
| **Maternal characteristics** |  |  |  |  |  |  |
| Mother’s age, years | 0.003 | 0.256 | 0.640 | 0.002 | 0.667 | 0.876 |
| Mother’s education | 0.000 | 0.709 | 0.886 | 0.012 | 0.297 | 0.876 |
| GDM | 0.000 | 0.986 | 0.986 | 0.000 | 0.900 | 0.900 |
| Preeclampsia | 0.003 | 0.238 | 0.640 | 0.002 | 0.702 | 0.876 |
| Histologic chorioamnionitis | 0.001 | 0.552 | 0.886 | 0.004 | 0.536 | 0.876 |
| **Infant characteristics** |  |  |  |  |  |  |
| Gestational age, weeks | 0.004 | 0.184 | 0.337 | 0.000 | 0.918 | 0.996 |
| Male sex | 0.015 | **0.009** | **0.020** | 0.060 | **0.017** | 0.187 |
| Apgar 5 min | 0.001 | 0.454 | 0.624 | 0.003 | 0.592 | 0.994 |
| Antenatal steroid | 0.000 | 0.703 | 0.859 | 0.001 | 0.808 | 0.994 |
| BPD≥moderate | 0.000 | 0.871 | 0.950 | 0.001 | 0.813 | 0.994 |
| IVH≥grade III | 0.030 | **<0.001** | **<0.001** | 0.002 | 0.679 | 0.994 |
| NEC≥stage 2 | 0.003 | 0.278 | 0.437 | 0.004 | 0.523 | 0.994 |
| PROM | 0.000 | 0.950 | 0.950 | 0.008 | 0.381 | 0.994 |
| Sepsis | 0.031 | **<0.001** | **<0.001** | 0.000 | 0.996 | 0.996 |
| TPN days | 0.033 | **<0.001** | **<0.001** | 0.030 | 0.094 | 0.345 |
| COVID-19 exposure | 0.042 | **<0.001** | **<0.001** | 0.047 | **0.036** | 0.198 |

GDM, gestational diabetes mellitus; BPD, bronchopulmonary dysplasia; IVH, intraventricular hemorrhage; NEC, necrotizing enterocolitis; PROM, premature rupture of membranes; TPN, total parenteral nutrition

**Supplementary table 8.** Neurodevelopmental outcomes following exposure to coronavirus disease 2019 in early childhood (0–5 years)

| **Authors (year)** | **Country** | **Population**  **(N)** | **Age at assessment** | **Type of assessment** | **Time points of exposure** | **Study period** | **Main outcome** |
| --- | --- | --- | --- | --- | --- | --- | --- |
| Huang et al. (2021) (1) | China | TDC  (N=6,054) | 6 M and 1 Y | ASQ-3, GDS | Assessment | Pre-pandemic (2015 to 2019)  Pandemic (March 1 to May 15, 2020) | The pandemic group had delayed fine motor and communication skills at 1 Y. |
| Imboden et al. (2021) (2) | United States | TDC  (N=1,024) | 6, 12, 18, 24, and 36 M | ASQ-3 | Assessment | Pre-pandemic (October 2018 to January 2019)  Pandemic (October 2020 to January 2021) | The pandemic group had impaired problem-solving abilities at 6M and communication skills at 6 and 12 M. |
| Sato et al. (2023) (3) | Japan | TDC  (N=1,227) | 3 and 5 Y | KIDS | Assessment | Pre-pandemic (November 2017 to October 2019)  Pandemic (December 2020 to November 2021) | The pandemic group showed developmental delays of 4.39 M. |
| Shuffery et al. (2022) (4) | United States | Children at high-risk  (N=255) | 6 M | ASQ-3 | Birth | Pre-pandemic (November 2017 to January 2020)  Pandemic (March 2020 to December 2020) | The pandemic group showed delayed gross motor and fine motor skills, and personal-social dysfunction at 6 M. |
| Giesbrecht et al. (2022) (5) | Canada | Children at high-risk  (N=3,742) | 1 Y | ASQ-3 | Assessment | Pre-pandemic (2016 to 2019)  Pandemic (April 2020 to April 2021) | The pandemic group had delayed communication, gross motor, and personal-social skills. |
| Scheiber et al. (2023) (6) | United States | Children at high-risk  (N=104) | 3 to 5 Y | CBCL | Assessment | Pre-pandemic (June 2019 to March 2020)  Pandemic (April 2020 to October 2020) | The pandemic group had increased internalizing and externalizing symptoms. |
| David et al. (2023) (7) | United States | VLBW  (N=340) | 4, 8, and 20 M at CA | BSID-III | Assessment | Pre-pandemic (January 2016 to December 2020)  Pandemic (March 2020 to April 2020) | The pandemic group showed a reduction in the cognitive and language scores at 20 M. |

High-risk children born to mothers with a history of genetic and developmental disabilities or preterm infants. COVID-19, coronavirus disease 2019; TDC, typically developing children; MSEL, Mullen Scales of Early Learning; ELC, early learning composite; VDQ, verbal developmental quotient; NVDQ, nonverbal developmental quotient; ASQ, ages and stages questionnaire; GDS, Gesell developmental schedules; VPT, very preterm; NICU, neonatal intensive care unit; CBCL, Child Behavior Checklist; IDAS, Inventory of Depression and Anxiety Symptoms; VLBW, very low birth weight; CA, corrected age; BSID, Bayley Scales of Infant and Toddler Development; KIDS, kindergarten infant development scale

**References**

1. Huang P, Zhou F, Guo Y, Yuan S, Lin S, Lu J, et al. Association between the Covid-19 Pandemic and Infant Neurodevelopment: A Comparison before and During Covid-19. *Front Pediatr* (2021) 9:662165. Epub 2021/10/26. doi: 10.3389/fped.2021.662165.

2. Imboden A, Sobczak BK, Griffin V. The Impact of the Covid-19 Pandemic on Infant and Toddler Development. *J Am Assoc Nurse Pract* (2021). Epub 2021/09/15. doi: 10.1097/jxx.0000000000000653.

3. Sato K, Fukai T, Fujisawa KK, Nakamuro M. Association between the Covid-19 Pandemic and Early Childhood Development. *JAMA Pediatr* (2023). Epub 2023/07/10. doi: 10.1001/jamapediatrics.2023.2096.

4. Shuffrey LC, Firestein MR, Kyle MH, Fields A, Alcántara C, Amso D, et al. Association of Birth During the Covid-19 Pandemic with Neurodevelopmental Status at 6 Months in Infants with and without in Utero Exposure to Maternal Sars-Cov-2 Infection. *JAMA Pediatr* (2022) 176(6):e215563. Epub 2022/01/05. doi: 10.1001/jamapediatrics.2021.5563.

5. Giesbrecht GF, Lebel C, Dennis CL, Silang K, Xie EB, Tough S, et al. Risk for Developmental Delay among Infants Born During the Covid-19 Pandemic. *J Dev Behav Pediatr* (2023) 44(6):e412-e20. Epub 2023/07/26. doi: 10.1097/dbp.0000000000001197.

6. Scheiber F, Nelson PM, Momany A, Ryckman KK, Ece Demir-Lira Ö. Parent Mental Health and Child Behavior During the Covid-19 Pandemic. *Child Youth Serv Rev* (2023) 148:106888. Epub 2023/02/28. doi: 10.1016/j.childyouth.2023.106888.

7. David J, Wambach CG, Kraemer M, Johnson TJ, Greene MM, Lee E, et al. Impact of the Covid-19 Pandemic on Early Intervention Utilization and Need for Referral after Nicu Discharge in Vlbw Infants. *J Perinatol* (2023). Epub 2023/07/07. doi: 10.1038/s41372-023-01711-7.
